# Tolerability and efficacy of induction Bacillus Calmette–Guérin for non-muscle invasive bladder cancer

**DOI:** 10.14440/bladder.2024.0051

**Published:** 2025-04-11

**Authors:** Mann Patel, Aravind Rajagopalan, Ellen M. Cahill, Kevin J. Chua, Rachel Passarelli, John Pfail, Sai Krishnaraya Doppalapudi, David Golombos, Thomas Jang, Vignesh T. Packiam, Saum Ghodoussipour

**Affiliations:** 1 Division of Urology, Department of Surgery, Rutgers Robert Wood Johnson Medical School, New Brunswick, New Jersey 08901, United States; 2 Department of Urology, School of Medicine, Yale University, New Haven, Connecticut 06510, United States; 3 Section of Urologic Oncology, Rutgers Cancer Institute of New Jersey, Rutgers Robert Wood Johnson University Hospital, New Brunswick, New Jersey 08901, United States

**Keywords:** Bacillus Calmette–Guérin, Adverse effects, Tolerability, Non-muscle-invasive bladder cancer

## Abstract

**Background:**

Intravesical Bacillus Calmette–Guérin (BCG) is the standard treatment for intermediate-risk, high-grade, and high-risk non-muscle invasive bladder cancer (NMIBC). However, it is associated with adverse effects, potentially causing treatment interruptions or discontinuation.

**Objectives:**

This study analyzed the tolerability and efficacy of induction BCG, with associated patient- and disease-related factors.

**Methods:**

A retrospective analysis was conducted on BCG-naive patients diagnosed with high-grade NMIBC, who received induction BCG at our institution between 2011 and 2021. Tolerability was defined as the completion of a 6-week induction course of BCG without treatment interruption or discontinuation. Multivariable logistic regression was performed to determine risk factors associated with the inability to tolerate treatment.

**Results:**

Induction BCG was given to 203 NMIBC patients, where 147 (72%) patients tolerated the treatment. Treatment interruptions occurred in 44 (22%) patients, while 12 (5.9%) patients discontinued the treatment. The median length of interruption was 1 week, primarily due to concerns about urinary tract infection (UTI) (*n* = 18, 41%) or gross hematuria (*n* = 5, 11%). No significant difference in 1-year recurrence rates was observed between those who tolerated BCG and those who did not (50% vs. 48%). Risk factors associated with the inability to tolerate induction BCG included male sex (odds ratio [OR] = 5.76, *p* < 0.01), hypertension (OR = 3.47, *p* = 0.02), and low pre-treatment hemoglobin levels (OR = 0.73, *p* = 0.03).

**Conclusion:**

Inability to tolerate BCG occurred in 28% of patients, with 5.9% experiencing discontinuation. Interruptions were short, mostly concerning UTI, and rarely leading to discontinuation. Poor tolerability was associated with male sex, hypertension, and low pre-treatment hemoglobin levels, highlighting critical targets for reducing the risk of BCG interruption or discontinuation.

## 1. Introduction

Bladder cancer is the most common cancer of the urinary tract and the fifth most common malignancy in the United States. In 2024, an estimation of 83,190 new cases and 16,840 deaths from bladder cancer is projected.^1^ Approximately 75% of patients with bladder cancer present with non-muscle invasive bladder cancer (NMIBC), which is associated with a high risk of recurrence or progression to muscle-invasive bladder cancer.^2^-^4^ Standard treatment for NMIBC involves transurethral resection of bladder tumor followed by intravesical administration of anti-cancer therapies.^5^ Bacillus Calmette–Guérin (BCG) has proven effective in treating NMIBC over the past several decades.^6^ According to the American Urological Association (AUA) guidelines, the current standard treatment for intermediate-risk, high-grade, and high-risk NMIBC patients consists of 6 weekly doses of induction intravesical BCG followed by 1 – 3 years of maintenance therapy.^3^,^4^ Survival rates after BCG instillations have been estimated at 81% and 74% for high-grade recurrence-free survival rates at 1 and 5 years, respectively. The estimated progression-free survival rates at 1 and 5 years are 97% and 92%, respectively.^7^,^8^

Despite its use as a treatment for NMIBC, patient responses to BCG vary significantly. Disease-free response rate ranges from 45% to 71% at 6-month post-BCG induction, with recurrence rates being 30% and 77%.^9^,^10^ Patients unresponsive to BCG – defined as those with persistent disease, disease progression, and disease recurrence despite adequate treatment or intolerance – experience poor outcomes, including decreased progression-free survival and increased rates of radical cystectomy.^9^,^10^ For these patients, further treatment options include radical cystectomy, enrollment in clinical trials, or alternative intravesical therapies.^5^

Individual tolerability of BCG immunotherapy can influence patient responsiveness. Adverse effects associated with BCG occur in up to 75% of patients and may include urinary frequency, urgency, nocturia, bladder pain, fever, chills, and hematuria.^2^ More severe side effects take place in <5% of patients, with disseminated infection from BCG found in <1%.^6^ Overall, treatment tolerability is improving over time. Prior studies reported treatment interruption rates of up to 40% due to adverse effects, whereas recent analyses showed interruption rates as low as 15% and discontinuation rates at 6%.^2^,^11^ Disease and clinicopathological-specific features, such as tumor grade and stage, as well as biomarkers, are strong predictors of BCG response.^12^,^13^ However, the predictive value of patient-specific factors remains poorly understood and under-researched.

We aimed to perform a contemporary analysis of the tolerability and efficacy of induction intravesical BCG immunotherapy for treating NMIBC. We hypothesized that many patients do not tolerate induction BCG and that certain patient-specific clinical and pathological factors predict tolerability. Furthermore, we hypothesized that patients with poor tolerability would exhibit higher rates of disease recurrence or progression than their counterparts with good tolerability.

## 2. Materials and methods

### 2.1. Data collection

This study included adult patients with BCG-naive, AUA intermediate, or high-risk NMIBC treated with induction BCG at the Rutgers Cancer Institute of New Jersey from January 2011 to July 2021.^3^ All data were retrospectively extracted from the electronic medical record, with project approval from the Rutgers University Health Sciences eIRB (Project# 2021002107). Relevant data collected included demographic information, medical comorbidities, pre-operative laboratory results, primary tumor characteristics (including grade and stage), and treatment course details. Treatment course details included the type of BCG strain used, the number of induction doses, reasons for treatment delays or discontinuations, reported adverse events (AEs), and utilization of maintenance therapy. BCG dose was classified as either 1/2, 1/3, or a full dose of 50 mg using the BCG TICE strain. Tolerance to induction was defined as the completion of a 6-week induction course of BCG without interruption or discontinuation. Inability to tolerate treatment included any interruption and/or discontinuation of the 6-week induction course of BCG. This definition of tolerability differs from other studies to more comprehensively identify predictors of unresponsiveness that may affect treatment continuity. Treatment toxicities were assessed based on the incidence of AEs and reasons for treatment delays or discontinuations. AEs were categorized according to the National Cancer Institute Common Terminology Criteria for AEs version 5 and were harvested through a retrospective review of patient records.

### 2.2. Statistical analysis

The characteristics of patients, including demographic, disease-related, and treatment-related variables, were summarized using medians and interquartile ranges for continuous variables, and frequencies for categorical variables. The distributions of baseline characteristics were compared using the Mann–Whitney U-tests for continuous variables and Chi-squared tests for categorical variables. Multivariable analysis was conducted to identify factors associated with BCG tolerability, controlling for patient age, sex, clinically-relevant variables such as glomerular filtration rate (GFR), albumin, tumor grade, and BCG dosage, and all statistically significant variables between groups. All analyses were performed using R-Studio with R Version 4.0.4. Comparisons were made using two-sided tests, with a *p* < 0.05 considered statistically significant.

## 3. Results

A total of 203 patients with NMIBC met the inclusion criteria (Table 1). The median age was 70 years, with a predominance of male patients (78%), White individuals (80%), and current or past smokers (72%). Tumor characteristics and comorbidities, including hypertension (60%) and diabetes (24%), are detailed in Table 1. According to the AUA guidelines, most patients (82%) had high-risk disease, and many presented with solitary tumors (57%) or high-grade tumors (88%). Recurrence or progression of tumors within 1 year occurred in 101 patients (50%).

**Table 1:** Patient- and disease-specific factors and tolerability of induction BCG (*n*=203)

Patient characteristic	Overall (*n*=203)	Tolerant (*n*=147)	Intolerant (*n*=56)	*p*-value
		
	Median (IQR)/*n* (%)	Median (IQR)/*n* (%)	Median (IQR)/*n* (%)	
Age	70 (63 – 78)	71.00 (63 – 78)	69 (64 – 78.25)	0.75
Sex				
Male	159 (78)	111 (76)	48 (86)	0.17
Female	44 (22)	36 (25)	8 (14)	
Hypertension	121 (60)	78 (53)	43 (77)	<0.01*
Hyperlipidemia	78 (38)	50 (34)	28 (50)	0.05*
Diabetes	49 (24)	29 (20)	20 (36)	0.03*
GFR	73.40 (56.10 – 86.70)	75.20 (59.55 – 89.10)	67.20 (53.60 – 86.10)	0.10
Albumin	4.30 (4.10 – 4.40)	4.30 (4.10 – 4.50)	4.10 (4.00 – 4.40)	0.05*
Hemoglobin	13.70 (12.70 – 14.70)	13.95 (12.90 – 14.70)	13 (11.65 – 14.03)	<0.01*
Stage				
Ta	80 (39)	63 (43)	17 (30)	0.14
T1	96 (47)	66 (45)	30 (54)	0.34
CIS	66 (33)	48 (33)	18 (32)	1.00
Tumor grade				
Low	28 (14)	19 (13)	9 (16)	0.72
High	179 (88)	130 (88)	49 (88)	1.00
Risk stratification				
Intermediate	37 (18)	29 (20)	8 (14)	0.49
High	166 (82)	118 (80)	48 (86)
BCG induction dose				
Full dose	197 (97)	144 (97)	54 (96)	1.00
1/3 Dose	6 (3.0)	4 (2.7)	2 (3.6)	
Maintenance BCG given	17 (8.4)	12 (8.2)	5 (8.9)	1.00
One-year recurrence				
Yes	101 (50)	74 (50)	26 (48)	0.50
No	68 (34)	46 (31)	24 (44)	
Unknown	13 (6.4)	9 (6.1)	4 (7.4)	

Note:

* Represents statistical significance (*p*<0.05). Abbreviations: BCG: Bacillus Calmette–Guérin; CIS: Carcinoma *in situ*; GFR: Glomerular filtration rate; IQR: Interquartile range.

A total of 147 patients (72%) tolerated the full treatment as scheduled. Among those who did not tolerate treatment, 44 patients (22%) completed the course with one or more interruptions, while 12 patients (5.9%) discontinued BCG altogether. The median length of treatment interruption lasted 1 week, primarily due to concerns of urinary tract infection (UTI) (*n* = 18, 41%) or gross hematuria (*n* = 5, 11%). Most patients who did not tolerate treatment received only one out of six doses (32%) before treatment interruption. Full doses of BCG were given to 198 patients, with 144 (72%) tolerating them, while 1/3 doses were given to six patients, with a tolerance rate of 67% (*n* = 4).

AEs are summarized in Table 2. Most reported AEs were classified as low-grade (grades 1 – 2). For patients who tolerated BCG treatment, the most common low-grade AEs included urinary symptoms such as frequency, urgency, or dysuria (17%); hematuria (11%); and UTIs (9.8%). In contrast, the most common low-grade AEs for patients who did not tolerate BCG included UTIs (36%), hematuria (11%), allergic reactions (3.6%), and difficulties with Foley catheter placement (3.6%). High-grade AEs (grades 3 – 5) occurred in 2.5% of patients who tolerated BCG and 5.6% of patients who did not tolerate it. Hospitalization was the primary high-grade AE reported among patients who tolerated treatment (2.5%). For those who did not tolerate BCG, frequently reported high-grade AEs included urosepsis (1.8%), hydronephrosis (1.8%), and coronary artery bypass graft surgery (1.8%).

**Table 2:** Adverse events and reasons for BCG delay or discontinuation

BCG induction completion rate	Number (%)	
BCG interrupted and completed	44 (22)	
BCG discontinued	12 (5.9)	
Length of BCG interruption (weeks) (median, IQR)	1 (1 – 2)	

**Reported AEs**		

**Low-grade AEs (Grade 1 – 2)**	**BCG tolerant patients (*n*=148) (%)**	**BCG intolerant patients (*n*=56) (%)**

Hematuria	23 (11)	6 (11)
Difficulty passing Foley	0 (0)	2 (3.6)
Inability to tolerate BCG instillation	0 (0)	1 (1.8)
Urinary symptoms	34 (17)	0 (0)
Urticaria/allergic reaction	0 (0)	2 (3.6)
Flank pain	3 (1.5)	0 (0)
UTI	20 (9.8)	20 (36)
Fever	9 (4.4)	1 (1.8)
Herpes zoster	0 (0)	1 (1.8)
High-grade AEs (Grade 3 – 5)		
Urosepsis	0 (0)	1 (1.8)
CABG	0 (0)	1 (1.8)
Hydronephrosis	0 (0)	1 (1.8)
Hospitalization	5 (2.5)	0 (0)
BCG shortage	0 (0)	1 (1.8)
Lost to follow-up	0 (0)	2 (3.6)
Unknown/other	0 (0)	1 (1.8)

Abbreviations: AE: Adverse events; BCG: Bacillus Calmette–Guérin; CABG: Coronary artery bypass graft surgery; IQR: Interquartile range; UTI: Urinary tract infection.

Patient characteristics were compared between those who tolerated and those who did not tolerate BCG induction (Table 1). Patients who did not tolerate BCG were more likely to have hypertension (*p* < 0.01), hyperlipidemia (*p* = 0.05), and diabetes (*p* = 0.03). Lower serum albumin and hemoglobin levels were also associated with BCG intolerance (*p* = 0.05 and *p* < 0.01, respectively). No significant difference was found in median GFR between the two groups, nor were there significant differences in tumor stage, risk level, or 1-year recurrence rates.

Univariable logistic regression was performed to identify risk factors independently predictive of an inability to tolerate induction BCG. Risk factors associated with higher chance of intolerability included hypertension (odds ratio [OR] = 2.88, 95% confidence interval [CI] [1.47, 6.00], *p* < 0.01), hyperlipidemia (OR = 1.96, 95% CI [1.05, 3.68], *p* = 0.03), diabetes mellitus (OR = 2.28, 95% CI [1.15, 4.50], *p* = 0.02), GFR (OR = 0.98, 95% CI [0.97, 0.99], *p* = 0.04), and pre-treatment hemoglobin levels (OR = 0.77, 95% CI [0.64, 0.92], *p* < 0.01) (Table 3). Tumor stage, pre-treatment risk level, and albumin contents were not found to be independent predictors of intolerability on univariable analysis. Multivariable logistic regression was performed using established factors, such as age and sex, alongside identified significant risk factors. This model identified that the male sex (OR = 3.50, 95% CI [1.34, 10.58], *p* = 0.02), comorbid hypertension (OR = 2.57, 95% CI [1.08, 6.49], *p* = 0.04), and lower pre-treatment hemoglobin levels (OR = 0.78, 95% CI [0.63, 0.96], *p* = 0.02) were the strongest predictors of BCG tolerability (Table 3).

**Table 3:** Univariable and multivariable logistic regression of potential risk factors associated with inability to tolerate induction Bacillus Calmette–Guérin (*n*=203)

Variable	Univariable		Multivariable	
	
	OR (95% CI)	*p*-value	OR (95% CI)	*p*-value
Age	1.01 (0.98 – 1.04)	0.46	1.00 (0.96 – 1.04)	0.89
Female	Ref		Ref	
Male	2.00 (0.90 – 4.91)	0.10	3.50 (1.34 – 10.58)	0.02*
Comorbidities				
Hypertension	2.88 (1.47 – 6.00)	<0.01*	2.57 (1.08 – 6.49)	0.04*
Hyperlipidemia	1.96 (1.05 – 3.68)	0.03*	1.32 (0.60 – 2.88)	0.48
Diabetes mellitus	2.28 (1.15 – 4.50)	0.02*	1.92 (0.87 – 4.19)	0.10
Pre-treatment laboratory results				
GFR	0.98 (0.97 – 0.99)	0.04*	0.99 (0.98 – 1.00)	0.35
Albumin	0.34 (0.10 – 1.08)	0.07		
Hemoglobin	0.77 (0.64 – 0.92)	<0.01*	0.78 (0.63 – 0.96)	0.02*
Tumor characteristics				
CIS	Ref			
Papillary (Ta or T1)	1.01 (0.53 – 1.99)	0.97		
High-risk	1.55 (0.71 – 3.79)	0.30		

Notes:

* Represents statistical significance (*p*<0.05); Ref denotes variables used as reference compared to their respective counterparts. Abbreviations: CI: Confidence interval; CIS: Carcinoma *in situ*; GFR: Glomerular filtration rate; OR: Odds ratio.

## 4. Discussion

We conducted an institution-specific retrospective study of BCG-naive patients diagnosed with intermediate-risk, high-grade, and high-risk NMIBC undergoing induction intravesical BCG, aiming to isolate factors associated with tolerability. Approximately 28% of patients did not tolerate BCG induction, with the most common reason for treatment discontinuation or interruption being concerns for UTI or hematuria. Risk factors associated with an inability to tolerate BCG included male sex, hypertension, and lower pre-treatment hemoglobin levels. Furthermore, there was no significant difference in the 1-year recurrence rate based on tolerability status.

Although intravesical BCG immunotherapy remains the standard therapy for treating high-risk NMIBC, it may fail in up to 40% of NMIBC patients.^5^,^6^,^14^,^15^ Our study found an overall tolerability rate of 72%, with recurrence rates being within previously reported ranges of 58% and 48% among patients who were BCG tolerant and intolerant, respectively, showing no statistically significant difference.

Existing literature reports that the most frequently occurring AEs are localized reactions, including dysuria, hematuria, lower urinary tract symptoms (LUTS), and epididymal-orchitis, affecting 50 – 75% of patients, while systemic presentations such as fever, general malaise, rash, and sepsis are less frequent, affecting 30 – 40% of patients.^11^,^16^-^19^ In our analysis, we observed higher rates of dysuria, hematuria, and UTIs; however, these AEs occurred at lower rates than previously reported. Specifically, hematuria was noted in 11%, LUTS in up to 17%, cystitis in 9 – 36%, and fever in 2 – 4% of patients, depending on tolerability. In comparison, retrospective studies and prospective trials evaluating outcomes during both induction and maintenance treatment found hematuria rates of 19 – 23%, cystitis ranging from 4 to 23%, LUTS in 12 – 23%, and fever in 8 – 13% of patients.^11^,^16^,^17^ Several factors may explain these discrepancies, including variability in study design, cohort size and selection (such as excluding patients who could not tolerate the first induction course), and non-standardized reporting of AEs. As a retrospective study, our findings are also subject to potential under-reporting.

In addition, due to variations in follow-up periods and the inclusion of maintenance treatment data in prior studies, our study uniquely focused solely on the induction period, which may not capture the AEs observed during longer follow-up periods. However, Brausi *et al*.^11^ reported a consistent distribution of side effects in a prospective clinical trial with a 3-year follow-up period.^11^ The study described BCG tolerance as dose-dependent, with 16% of study participants experiencing delays or interruptions during both induction and maintenance treatment, and 6.2% discontinuing treatment, primarily within the 1^st^ year.^11^ In contrast, our study, which assessed the tolerance of induction treatment only, reported a comparable discontinuation rate (5.9%) but a slightly higher interruption rate (22%), which may be attributed to the differing follow-up periods.

Interestingly, many studies have not stratified AEs between BCG-intolerant and BCG-tolerant patients beyond the overall frequency of local or systemic side effects either. In our analysis, BCG-intolerant patients suffered from higher rates of UTIs and similar rates of hematuria as the most common reasons for treatment interruption. However, the intolerant patients had a lower frequency of fever with no reports of urinary symptoms, which may reflect under-reporting. It is clinically relevant to note that there was no identifiable pattern among the 12 patients who discontinued treatment. Reasons for discontinuation varied significantly and included urosepsis, hematuria, hydronephrosis, previous medical history (e.g., coronary artery bypass grafting), herpes zoster, and non-medical reasons, such as the ongoing BCG shortage or an inability to tolerate physical instillation. This underscores the difficulty in distinguishing between treatment interruption and full discontinuation in real-world settings based on AEs while characterizing overall tolerability.

While the prevalence of BCG intolerability and associated AEs is well documented, the underlying risk factors of intolerability remain poorly understood. Disease severity has been reported to contribute to worse prognoses, but it does not appear to affect initial tolerability or treatment completion. On the other hand, the impact of treatment dose and type has yielded mixed results.^17^,^20^,^21^ Our findings also indicate no significant influence of tumor or treatment characteristics on tolerability, highlighting the necessity to examine patient-specific characteristics. In this study, the inability to tolerate BCG was associated with male sex, hypertension, and lower pre-treatment hemoglobin levels.

Bladder cancer exhibits a gender disparity, with lower incidences but more aggressive and advanced presentations in females, leading to notable variability in prognoses.^22^-^26^ However, the sex-mediated distinctions in treatment tolerability are not well understood. Our study indicates that males are more likely to be BCG-intolerant. A retrospective study by Fadel *et al*.^22^ investigated sex-based differences in NMIBC patients and reported similar treatment completion rates for both sexes when considering more than five induction and maintenance BCG doses.^22^ In contrast, our study extended the definition of tolerability to include treatment interruptions and delays, suggesting a sex disparity in treatment tolerability. This difference may be attributed to obstructive urinary symptoms or the administration of BCG through catheterization, as concerns about hematuria and UTI are the primary reasons for interruptions. While female sex is a known risk factor for BCG-associated bacteriuria and catheter-associated UTIs, there is still a higher incidence of catheter-associated UTIs and significantly more catheter-associated hematuria among males.^27^,^28^ Increased susceptibility to UTIs and hematuria diminishes tolerability due to an elevated risk of interruption. In addition, biological differences in pharmacokinetics, hepatic metabolism of carcinogens, and sex steroid hormone synthesis pathways, factors known to affect prognoses, may also influence tolerability in ways not yet elucidated.^23^,^29^,^30^

We also identified hypertension and lower pre-treatment hemoglobin levels as predictors of BCG intolerability, suggesting that patients with pre-existing comorbidities may exhibit reduced tolerance. Prior studies have reported an increased risk of bladder cancer and recurrence; however, hypertension has not been previously identified as a predictor for intolerability.^31^-^33^ Hypertension is associated with vascular alterations, insufficient regional blood supply, elevated inflammation or oxidative stress, and neoantigen production – all of which may decrease the tolerability of live attenuated bacterial therapy.^31^,^34^ Moreover, hypertension is linked to various comorbidities within metabolic syndrome; the BCG-intolerant cohort showed higher prevalences of hyperlipidemia and diabetes mellitus. Metabolic syndrome not only increases the risk of bladder cancer but also contributes to LUTS and impaired immunity.^34^ Furthermore, pre-treatment hemoglobin levels, a widely used predictor of oncological treatment, are linked to an increased risk of aggravated AEs, including UTIs.^35^,^36^ Inferior baseline health may attenuate immune responses, contributing to BCG intolerability.

Our study did not find a difference in 1-year recurrence rates between patients who tolerated BCG and those who did not. A retrospective study by Nummi *et al*.^6^ previously reported virtually doubled recurrence rates associated with treatment interruption. However, the study defined recurrence occurring after 3 months or progression at any time during induction or maintenance treatment.^6^ While Nummi *et al*.^6^ included patients treated for recurrent tumors, our study focused on the 1-year period following induction and excluded patients with prior BCG therapy. Thus, discrepancies likely stem from nuances in study design and cohort-specific patient, disease, and treatment characteristics.

Particularly, disease-specific characteristics, such as tumor stage, multiple recurrence history, and presence of concomitant carcinoma *in situ* (CIS), have been shown to affect rates of recurrence and disease progression.^2^,^37^ Although our study did not find significant differences in these factors based on tolerability, it included a relatively higher prevalence of CIS, which is associated with increased recurrence rates as high as 30 – 50%, as reported by prior studies.^2^,^37^ In addition, the use of maintenance BCG therapy is another nuanced factor. The current guidelines recommend maintenance therapy following induction due to its proven effectiveness in reducing recurrence risk and delaying disease progression.^2^,^37^ However, only a small subset of patients (8%) in our cohort received maintenance BCG, which may have impacted recurrence rates. As most recurrences and disease progression occur within the first 5 years post-treatment, a longer follow-up period may provide further insights into these dynamics.^2^

Most importantly, our findings enhance confidence in the continued reliance on BCG immunotherapy and the management of its side effects. We demonstrated that 1 – 2-week delays due to concerns about hematuria or UTI do not compromise immediate efficacy, allowing adequate time for symptom management. In addition, we found that reduced BCG doses, necessitated by a BCG shortage during our study period, did not impair tolerability or 1-year efficacy. Notably, only 3% of patients in our cohort received less than a full treatment dose, which may limit direct comparisons. Nonetheless, our findings align with existing literature indicating that reducing doses does not alter efficacy and may even decrease toxicity.^17^,^20^,^21^

Identifying predictors of treatment tolerability facilitates the development of targeted pre-treatment strategies for high-risk patients. For example, pre-treatment risk stratification based on disease- and patient-specific factors, such as hypertension, anemia, and male sex, can guide initial interventions. While these factors often reflect poor baseline health, managing hypertension and improving hemoglobin levels before and during treatment may reduce the incidence of AEs. In addition, proactive management of BCG instillations in male patients, who are at an increased risk of catheter-associated UTIs, and early intervention for urinary symptoms, potentially through antibiotic prophylaxis, could enhance tolerability. However, further research is necessary to validate these strategies in higher-risk populations.

There are several limitations to our study. As a single-institution retrospective study, our data may be limited in generalizability, and we cannot account for variations in care and clinical documentation that could lead to discrepancies in treatment course determination and AE reporting. Data collection across multiple electronic medical records may also contribute to under-reporting of AEs. Furthermore, the study period coincided with a national BCG shortage, leading to modified treatment strategies primarily involving reduced dosing (1/3 standard dose) and inconsistent real-world data suggesting increased rates of treatment interruptions and recurrence rates.^38^-^40^ While reduced dosing did not differ significantly between groups, this may have resulted in fewer or reduced doses of BCG for our patients, potentially overestimating interruption rates and contributing to the low utilization of maintenance dosing. In addition, nuances in cohort selection, such as increased rates of CIS, may mirror increased referral of higher-risk patients to our tertiary comprehensive cancer center. Finally, our follow-up period was shorter than those of previous studies due to our focus on the induction phase. Thus, it may be insufficient to evaluate the long-term impact of the identified risk factors as well as long-term efficacy and recurrence rates beyond the scope of this study.

## 5. Conclusion

In this cohort, 22% of patients undergoing induction BCG immunotherapy experienced treatment interruption and 5.9% discontinued treatment. We found that an inability to tolerate BCG rarely led to treatment discontinuation or significant differences in 1-year recurrence rates. Predictive factors for intolerability included male sex, hypertension, and lower pre-treatment hemoglobin levels, with concern about UTI being the most common reason for interruptions. Future efforts will focus on extending the follow-up period, assessing efficacy rates and treatment failures, and expanding the clinical characteristics included in the regression model. Ultimately, the current understanding remains inconsistent, highlighting the need for prospective studies to validate these findings.

## Data Availability

The raw data from this study will be made available by the authors upon reasonable request.
